# The Doctor and His Relation to Society

**Published:** 1908-07

**Authors:** J. C. Olmsted

**Affiliations:** Atlanta, Ga.


### Journal-Record of Medicine

Successor to Atlanta Medical and Surgical Journal, Established 1855, and Southern Medical Record, Established 1870.

**OWNED BY THE ATLANTA MEDICAL JOURNAL CO.**

**Published Monthly.**

*Official Organ Fulton County Medical Society, State Examining Board, Presbyterian Hospital, Etc.*

EDGAR G. BALLENGER, M. D., Editor.
BERNARD WOLFF, M. D., Supervising Editor.
A. W. STIRLING, M. D., C. M., D. P. H., J. S. HURT, B. Ph., M. D., Associate Editors
E. W. ALLEN, Business Manager.

COLLABORATORS

DR. W. F. WESTMORLAND, General Surgery.
F. W. McRAE, M. D., Abdominal Surgery.
H. F. HARRIS, M. D., Pathology and Bacteriology.
E. B. BLOCK, M. D., Diseases of the Nervous System.
MICHEL HOKE, M. D., Orthopedic Surgery.
CYRUS W. STRICKLER, M. D., Legal Medicine and Medical Legislation.
E. C. DAVIS, A. B., M. D., Obstetrics.
E. G. JONES, A. B., M. D., Gynecology.
R. T. DORSEY, Jr., B. S. M. D., Medicine.
L. M. GAINES, A. B., M. D., Internal Medicine.
J. N. LeCONTE, M. D., Diseases of the Stomach and Intestiness.
L. B. CLARKE, M. D., Pediatrics.
EDGAR PAULIN, M. D., Opsonic Medicine.
R. R. DALY, M. D., Medical Society.
A. W. STIRLING, M. D., Etc., Diseases of the Eye, Ear, Nose and Throat.
BERNARD WOLFF, M. D., Diseases of the Skin.
E. G. BALLENGER, M. D., Diseases of the Genito Urinary Organs.

VOL. X. JULY, 1908. NO. 4

### THE DOCTOR AND HIS RELATION TO SOCIETY.*

BY J. C. OLMSTED, M. D., ATLANTA, GA.

Ladies and Gentlemen, Doctors of the Graduating Class: In the countries of the old world, where the order of Knighthood exists, when one is admitted to this order, his sovereign touches him on the shoulder with the flat of a sword, and he is said to receive the "accolade," which confers upon him the rank of "Sir Knight." On this occasion, gentlemen of the graduating class, your alma mater in presenting you with your well earned diplomas, confers the "accolade," which admits you, and consecrates you to the service of a profession, nobler, and more ancient by far, than any order of Knighthood. A profession whose beneficent history reaches so far back in the annals of our race, that it is

*Annual address at the Commencement of the Atlanta School of Medicine, April 22, 1908.

lost in tradition, amidst the shadows, and night, of a remote antiquity. You have indeed a right to beproud of your deploma as "Doctor in Medicine." It comes as the reward of laborious years of diligent study under the direction of accomplished, experienced, and painstaking teachers, who have instructed you in those various branches of science, upon whose broad and deep foundations your profession rests, founded as upon a rock. You now stand upon the treshold of your career, and this is truly your "commencement," for you are now to go forth, to apply the scientific principles which you have received, to that most difficult Art, the practice of Medicine. I shall on this occasion, speak as briefly as I can, on the Doctor in his relations to society; using this term in its broad sense of community, and I may here say with Burns,

"But how the subject theme may gang, let time and chance determine;
"Perhaps it may turn out a song, perhaps turn out a sermon."

I will not promise to stick closely to my text, as good preachers are said to do; I may turn up in unexpected places, but so does our friend the Doctor, and hence I am sure to be logical! I confidently assume in the premises, that the Doctor is as interesting as he is an important character in the community. The presence here to night, of this large audience, is one guarantee of the truth of this statement. Woman deigns to grace the occasion with her presence, and to smile encouragement, auspicate success, on your entrance into the noblest of professions; not excepting perhaps, the Christian ministry; for in the sacredness of its duties and obligations, in devotion to duty, and in large self-sacrifice, it is in my humble opinion, "second to none!"

Have you my younger brothers, who aspire to be ministers in this temple of medicine, reflected upon the serious responsibilities which you invite? Have you considered the lofty aims, the self-sacrifice for the good of others, the purity of mind and heart, which should characterize him who enters here? Without these, we will never be worthy of that title of highest honor, which in ages past was bestowed upon members of our profession, when they were called "the Divine" or "God-like men." To be worthy of the noble character which is here implied, should indeed be the high aim of us all; and no lower standard should suffice. Of the profession of medicine it has been truly said, that it is "the noblest of professions, and the meanest of trades." That the "trade" spirit is abroad in certain quarters of the profession, is not to be denied; and is only an admission that the medical profession, like everything else human, has been contaminated by the dollar chasing craze of this age; but not yet have "the money changers" taken entire possession of the "sacred temple of Aesculapius!" There yet remains a goodly host of its disciples, with whom its primitive doctrines, and traditions, are yet vital principles, and perennial truths; such as these, uphold their noble art, in all its primal beauty, and redeem it from the withering blight, of mere self-seeking gain. I am not urging an impossible romantic altruism, that the doctor has a right to expect compensation for his work, is not in antagonism to the foregoing; nor does this deny the claims of true charity; but he too must live, and even St. Paul declared that "they who preach the Gospel, should live of the Gospel." Yes, the doctor, and the preacher must live, although some people in their treatment of both, seem to have forgotten this important truth! The doctor may incidentally grow rich by his profession; few of us, are exposed to this danger! But this will ever be a secondary consideration, with the true hearted physician. Never will he prostitute his noble art, to this ignoble aim, and end. His wealth is for other than this; it is a "treasure" of good deeds, noble efforts, lofty aspirations, in an impregnable character, which "neither moth" of greedy gain, "nor rust" of neglected worth, can "corrupt;" nor "thieves," of time serving policies, "break through, and steal!"

The practice of medicine is as truly an art, as painting, music, literature, etc. Like these arts, it has of course certain scientific principles, and aspects; but it is more of an art, than that exact science, which some would claim it to be. And so in the main, it must ever be from the nature of the conditions and circumstances, by which it is limited, and under which its processes must be conducted. These being variable, it is hence impossible to limit it, within the "hard and fast rules," of a "fixed science." He is the master of this art, who best understands the peculiar conditions, and features of the case before him, and hence is best prepared to meet its requirements; just as the painter mixes his colors, and blends them to express the light, and shadows and objects, in the particular landscape before him, even so the physician, considers the particular features of the individaul case before him, and adapts his remedies, to meet its especial requirements. Among the laity generally the impression prevails, that diseases are treated according to their names: Such and such a thing "is good for pneumonia" or "typhoid fever," etc. I have seen this error of treating diseases by their names, committed by some physicians, that important factor, the individual with the disease, being apparently ignored! With the laity this impression is I believe fostered by the gorgeous rhetoric of the multitudinous "patent medicines," so dear to the public heart. But dare I impugn the magic virtues of "Peruna," "Paines Celery Compound," "Carter's Little Liver Pills" and "Brown's Iron Bitters?" These, and many others, all "vouched for," mostly by venerable clergymen, with affidavit faces, or benevolent, grand, motherly-looking old ladies, to question whose veracity, would almost seem impossiblé! The physician who treats all cases of the same disease, in exactly the same manner, will commit as egregious blunders as would the painter, who should apply the same blending of colors, in the same manner, without regard to the varying effects of light, and shade, and perspective, etc., in different landscapes. In the case of the painter, a "botch of a picture" is the result, in that of the doctor, a grave may be filled. The public often think it strange, to hear physicians speak of the fascination which the study and practice of their art possesses for them. Yet by the true physician, it cannot be imagined, that the arts of music, poetry, painting, etc., afford an enjoyment more keen than his. And surely the doctor needs this enthusiasm, for in the words of the old song, he often has "a hard road to travel," with days of trial, and often nights "devoid of ease," and it may be at times ingratitude as the reward of his patient, well directed efforts, he must find his consolation and happiness in the higher realms of his noble calling, in its intellectual triumphs, and the consciousness of duty well performed. The doctor in his relations to society, should have a many sided mind, that is he should be a man of liberal culture, and informed on many and varied subjects; his opinion being not infreqeuntly called for, an subjects of general interest to society. He should be capable of adapting himself to that "all sorts and conditions of men," high or low, rich or poor, learned or unlearned, with whom he is brought in contact.

Think of it! To what a variety of characters, good, bad, and indifferent, of minds weak, strong or mediocre, sane, partially sane, and insane, does the doctor have to adapt himself? He must listen with a grave face, and respectful bearing to the oft repeated "tale of woe" of the hypochondriac, whose ruddy cheek and general good health, belie his utterly impossible symptoms. or patiently investigate "the most peculiar symptoms doctor," of the imaginative lady, who is sure that she has a cancer! He must attempt to live through his many and varied experiences with the hysterical lady, whom perhaps wealth and idleness, allied to a naturally peevish disposition, have combined to form this plague of the doctor's life. He must preserve his equanimity of temper, when under fire of the old "Colonel," who, furious from the pain of "suppressed gout," and unsuppressed ill-temper, is disposed to hold the doctor, and not the wine he has drunk, personally responsible for his condition. From giving a "plain talk" to some in need of that discipline, he must approach the frail, nervous, and timid woman, whose life is to be counted by days, it may be hours. Not only caring for their physical ailments, but as their trusted friend, and wise councillor, he bears in his bosom, the secret sorrows of many a household. To the doctor the community looks, for those directions, and well planned measures, by which its health is preserved, and pestilence is warded off. And yet, how often in his efforts, to have wise laws enacted for the protection of the community from contagious, and infectious diseases, is he thwarted by the ignorance, or culpable indifference of selfish politicians bent only upon their schemes of personal aggrandizement! These so-called "Solons" (Save the mark!) whose often foolish performances in the legislature, cumber the statute books with worthless "laws," forgotten as soon as passed, and promptly buried in well merited oblivion! And yet the doctor was seeking to protect the community, by efforts to eliminate disease, and hence in large measure, to eliminate himself, by preventing, rather than curing disease!

Is there any other calling, or profession on earth, with a financial interest connected with it, whose highest aim is to limit the sphere of its operations, and personal interests? But occasionally there comes along, some shrewd, witty, and cynical so-called "philosopher," chiefly engaged in making money, and airing a loose morality, and who in a brilliant "lecture" gravely informs the public, that "the doctors are parasites!"

This of a profession, which not to speak of its many achievements, in relieving suffering humanity in other fields of disease, has practically abolished that hideous disease "small pox," where it's advice has been taken; which has done much to circumscribe the ravages of cholera, and is now engaged in a hopeful struggle, with the "plague" of the East, and Tuberculosis, the world over; and which last, but not least, to us of the South, has banished from our shores, that dread scourge "yellow fever," which used to depopulate cities, paralyze their commerce, fill their cemeteries with its victims, and send the panic-stricken inhabitants, flying for protection to other sections of the country; poor, distracted wanderers, often treated as most unwelcome guests! Think of the suffering, and death, not to mention the immense financial loss, which was involved in a visitation of "yellow fever!" All of this has now passed away, like some hideous vision of the night. By what, and whom, has this change been accomplished? By the doctors, those heroic men, who in discovering the cause of the diffusion of this disease, risked their lives, and in some instances lost them, in personal experiments upon themselves, to prove the source of contagion! Nor should we forget, that army of the dead of our profession, who lost their lives in fighting this disease, and who lie in the cemeteries of Savannah, Mobile, New Orleans, Vicksburg, Granada, Holly Springs, Chattanooga, and Memphis, with its "Doctor's Street," as they call that portion of the cemetery where they rest! This is a list of "battle fields," where fell many who left happy homes, in healthy localities, to aid their over-worked brethren in the cause of humanity, and who returned no more! Their claims to grateful remembrance and immortal fame, forgotten, perhaps, in the lapse of years; voiced it may be only, by the complaining night wind, as it murmurs through the trees that shadow their humble patriot graves! Surely for such, if for any, should the "monument of enduring bronze, and everlasting marble," uplift it's stately shaft, to greet the earliest light of the morning, and reflect the splendors of parting day, in a golden glow of immortal memories! As I think of our profession in its high service to humanity, I love to associate the doctor, with that beautiful legend, which comes to us from the far, and mystic East, in which the poet tells us how—

"Abou Ben Adhem, may his tribe increase!
"Awoke one night, from a deep dream of peace;
"And saw within the moonlight of his room,
"Making it rich, and like a lily in bloom,
"An angel, writing in a book of gold.
"Exceeding peace, had made Ben Adhem bold;
"And to the presence in the room he said,
"What writest thou?" The vi sion raised it's head,
"And with a look, made of all sweet accord,
"Answered, "the names of those that love the Lord."
"And is mine one?" Said Abou. "Nay, not so"
"Replied the angel, Abou spoke more low,
But cheerly still, and said "I pray thee then
Write me as one that loves his fellow men!"
"The angel wrote and vanished, the next night
It came again, with a great wakening light,
"And showed the names, whom love of God had blessed,
And lo! Ben Adhem's name led all the rest!"

